# Supplementary figures and images for: Purification of plant-derived anti-virus mAb through optimized pH conditions for coupling between protein A and epoxy-activated beads
Source: PeerJ. 2019 May 21;7:e6828. doi: 10.7717/peerj.6828 (PMC6534112; doi:10.7717/peerj.6828)

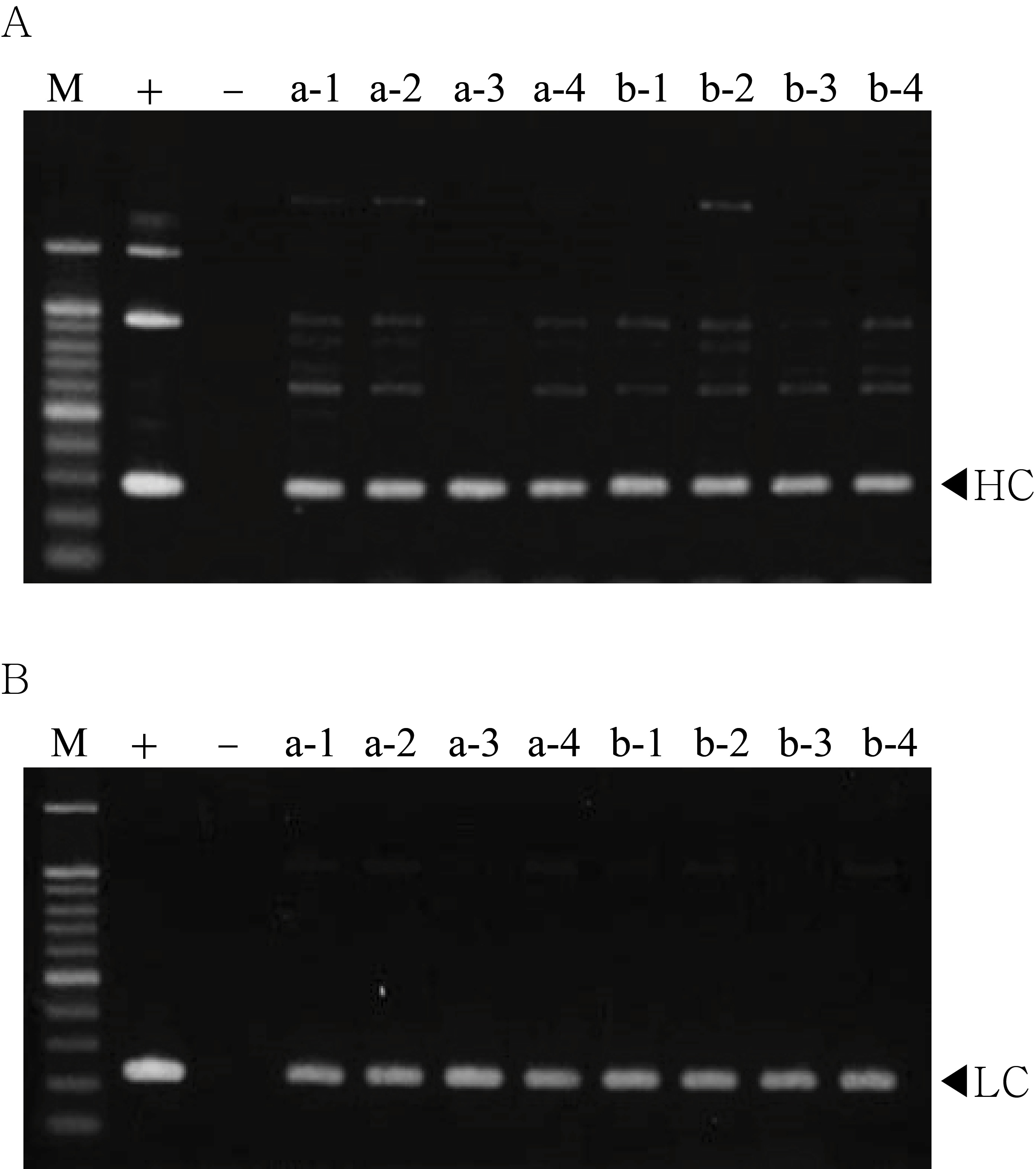

Supplement: Supplemental Information 1 — The genomic DNA fragments were extracted from fresh leaf tissue, amplified, and separated on a 1% agarose gel using electrophoresis. Positive control (+), pBI mAb SO57 vector in DH5α competent cells, negative control (−), and non-transgenic tobacco plant (NT). [file peerj-07-6828-s001.jpg]

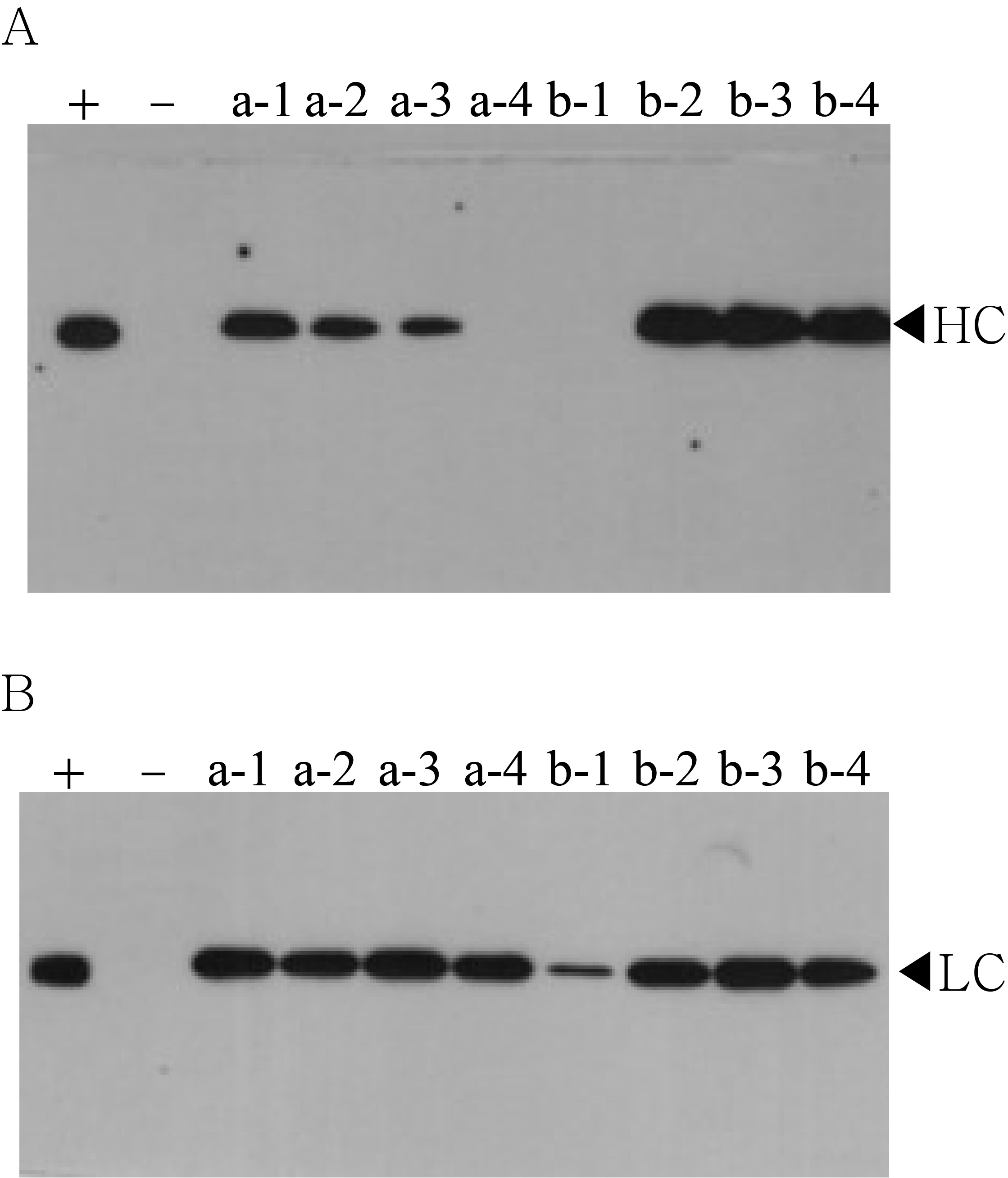

Supplement: Supplemental Information 2 — HC (50 kDa) and LC (25 kDa) were detected with HRP-conjugated goat anti-human IgG Fc- or IgG F(ab′)2-specific antibodies, respectively. Lane 3–10, T1 transformants putatively expressing anti-rabies mAbPSO57. Positive control (+); human rabies immunoglobulin(HRIG) and negative control (−); non-transgenic tobacco plant (NT). [file peerj-07-6828-s002.jpg]

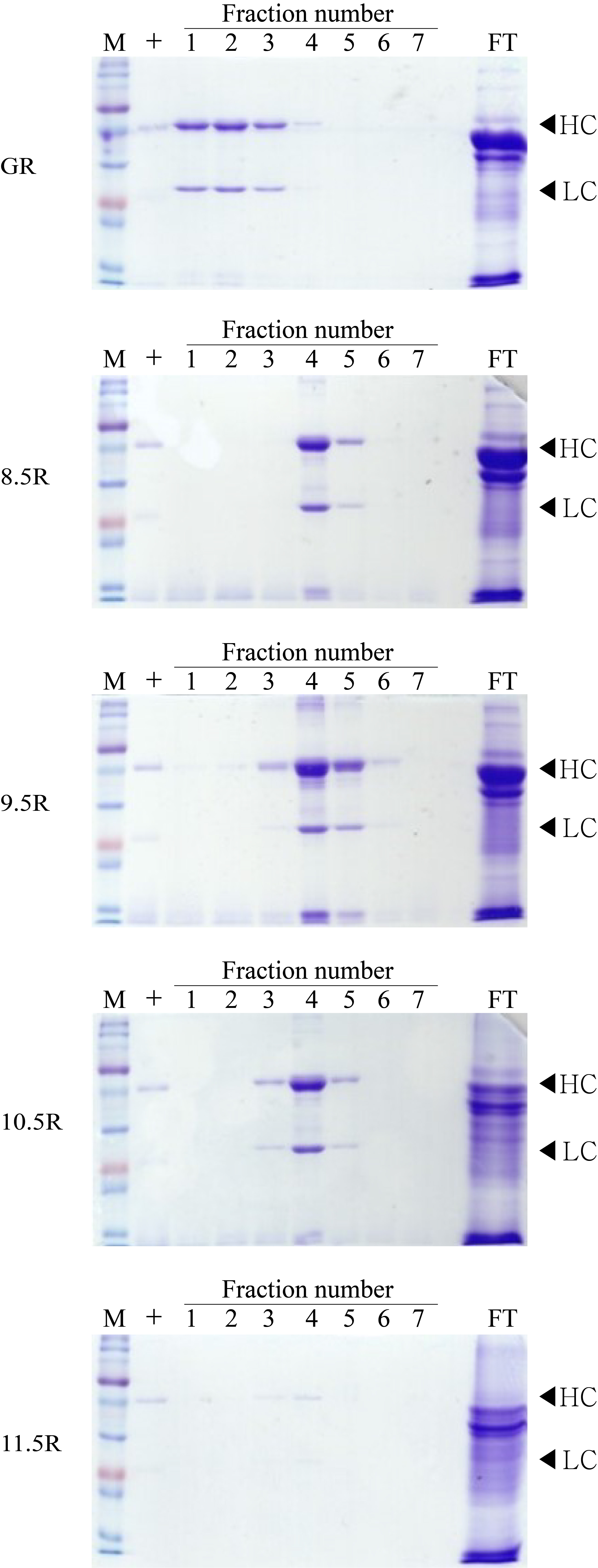

Supplement: Supplemental Information 3 — Lane 1, protein marker; Lane 2, positive control (+), human rabies immunoglobulin (HRIG); Lane 3–9, eluted fractions of the purification F1–F7, respectively; Lane 11, flow through; HC, heavy chain of mAbP; LC, light chain of mAbP. Epoxy-activated agarose beads were coupled to protein A under the pH conditions of 8.5, 9.5, 10.5, and 11.5 (8.5R, 9.5R, 10.5R, and 11.5R, respectively). Commercial protein A resin (GR) (GE Healthcare, Uppsala, Sweden) was used as a positive control. [file peerj-07-6828-s003.jpg]
